# Cellular Stress Impact on Yeast Activity in Biotechnological Processes—A Short Overview

**DOI:** 10.3390/microorganisms11102522

**Published:** 2023-10-09

**Authors:** Madalina Postaru, Alexandra Tucaliuc, Dan Cascaval, Anca-Irina Galaction

**Affiliations:** 1 Department of Biomedical Science, Faculty of Medical Bioengineering, “Grigore T. Popa” University of Medicine and Pharmacy of Iasi, M. Kogălniceanu 9-13, 700454 Iasi, Romania; anca.galaction@umfiasi.ro; 2 Department of Organic, Biochemical and Food, “Cristofor Simionescu” Faculty of Chemical, Engineering and Environmental Protection, Engineering, “Gheorghe Asachi” Technical University of Iasi, D. Mangeron 73, 700050 Iasi, Romania; alexandra.tucaliuc@academic.tuiasi.ro (A.T.); dan.cascaval@academic.tuiasi.ro (D.C.)

**Keywords:** *Saccharomyces cerevisiae*, yeast fermentation, oxidative stress, ethanol stress, osmotic stress, cell response

## Abstract

The importance of *Saccharomyces cerevisiae* yeast cells is known worldwide, as they are the most used microorganisms in biotechnology for bioethanol and biofuel production. Also, they are analyzed and studied for their similar internal biochemical processes to human cells, for a better understanding of cell aging and response to cell stressors. The special ability of *S. cerevisiae* cells to develop in both aerobic and anaerobic conditions makes this microorganism a viable model to study the transformations and the way in which cellular metabolism is directed to face the stress conditions due to environmental changes. Thus, this review will emphasize the effects of oxidative, ethanol, and osmotic stress and also the physiological and genetic response of stress mitigation in yeast cells.

## 1. Introduction

Yeasts are microorganisms known for their role in fermentation processes. In other words, they transform complex substances from the environment into totally new ones that are needed in various fields. However, along the way, the microorganisms face several types of stresses, starting with osmotic stress, and as the food in the environment is consumed, stress occurs due to the lack of nutrients and the increasing amount of resulted ethanol [1]. The most important yeasts used in industry are the *Saccharomyces cerevisiae* species. But using them so intensively exposes them to a series of factors which can affect their activity during the fermentation process. Extreme values of pH or temperature, lack of nutrients in the environment, and metabolic reaction products are just a few examples. All the elements presented lead to different types of stress, such as oxidative, ethanol, saline or osmotic [2].

All the stress formed in the culture medium produces harmful effects on the yeast cells. As a result, their growth and metabolism are slowed down, and the lag phase is often extended. To prevent these events, at the cellular level, the processes of transcription regulation or stimulation of certain genes take place. Such genes encode both proteins and enzymes, which act in such a way as to improve the microorganism’s tolerance to stress [1,3]. Yeast cells can respond in a non-specific way to environmental conditions, so that their development is not disturbed during the time required for a specific response, through an evolutionary adaptation, namely the general stress response [4].

This paper emphasizes some types of cellular stress acting on *Saccharomyces cerevisiae* species. It also evaluates how *S. cerevisiae* cultures use their defense mechanisms to increase their tolerance to stressors. Understanding the aspects related to the action of cellular stress on yeasts and their response is important from a biotechnological point of view, being helpful for the optimization of industrial fermentation processes. This can reduce the amount of yeast cells used, and increase the quality for the final products.

## 2. *Saccharomyces cerevisiae* Species

*Saccharomyces cerevisiae* is a unicellular eukaryotic microorganism with a wide distribution in trees, soil, and fruits. Research has revealed that it can also be located in the urinary, respiratory or gastrointestinal tracts of healthy individuals [5,6,7,8,9,10,11,12,13]. As a facultative anaerobic microorganism, it is able to develop both in the presence and absence of oxygen. Depending on the oxygenation conditions, the yeast cell uses different biochemical mechanisms. Thus, in the absence of oxygen, but with a carbohydrate substrate, the energy required for cell development is obtained through glycolysis. In the presence of molecular oxygen and in the absence of sugars, respiration takes place at the mitochondrial level, with energy being obtained through oxidative phosphorylation [14].

In biotechnology, *S. cerevisiae* has been used for several decades to obtain alcohol by fermentation in the beer and wine industries, helping the dough rise during the baking process, and also plays a notable role in obtaining biofuels [15]. In terms of its widespread use for ethanol production, *S. cerevisiae* is very advantageous, as it has a non-pathogenic nature, has the ability to grow on inexpensive substrates, and to withstand high concentrations of alcohol (up to 10% *v*/*v*) [16]. *S. cerevisiae* could be a suitable option for obtaining other compounds, such as butanol (up to 2.5 mg/L with a 2% galactose carbon source, using ESY7 strain which overexpresses the native thiolase and HbCoA dehydrogenase), but there are still obstacles to overcome, in terms of expressing heterologous biosynthetic pathways [16,17].

Over the years, researchers were able to sequence its entire genome, discovering about 6000 genes. The data acquisition process was carried out through “omic” technologies, molecular biology techniques and traditional biochemistry. All information is currently included in the “*Saccharomyces* Genome Database” [18,19,20,21,22,23,24]. Yeast cell morphology has a relatively simple ellipsoidal shape and the sequences of genome leads to the easy production of mutants. A database for the morphological characterization of *S.cerevisiae* (SCMD) associates individual mutants of the *S. cerevisiae* genome with secondary gene annotation and with protein sequences [25]. Research on intracellular organelles of *S. cerevisiae*, specifically mitochondria, has shown that the most frequently occurring mutant is the respiratory deficiency (‘petite’) mutation, in which the mitochondria are incapable of synthesizing certain proteins and become partially unable to function aerobically. Consequently, the yeast cells can no longer metabolize non-fermentable carbon sources, like lactate, ethanol or glycerol. Furthermore, the cell becomes less tolerant to stress factors, such as ethanol and osmotic pressure [26].

*S. cerevisiae* grows in colonies when in adequate conditions, but under different stressors, yeast cells can alter their growth pattern to produce complex structures leading to a change in colony morphology and also at a genetic level. Therefore, different genes were identified to be involved in complex colony morphology, components of MAP kinase cascade and the Ras-cMAP-PKA pathway, which are the most researched pathways in eukaryotic organisms. Under different nutrient conditions, some strains develop complex colony morphology morphotypes, included in the following categories: spokes (OS17 strain), concentric rings (YJM224 strain), lacy (YJM311 strain), coralline (NKY292 strain), mountainous (PMY348 strain), and irregular (BY4743 strain) [27].

The importance of *Saccharomyces cerevisiae* yeast cells is given by certain proteins that are considered to be similar to human ones. Thus, yeast cells are used in various studies to analyze biochemical processes that have similar effects in human cells, like aging, cellular stress, and drug screening [13,28,29,30,31]. Another role for this microorganism is its involvement in the molecular mechanisms of response to oxidative stress and in the correlation between reactive oxygen species and aging processes at the cellular level. *S. cerevisiae* is both genetically and reproducibly safe to grow due to short-lived generations of between 1.5 and 3 h. From this point of view, the microorganism is intensively used as an experimental model for the study of certain genetic and cellular aspects [32].

## 3. Types of Cellular Stress

**Figure 1:**
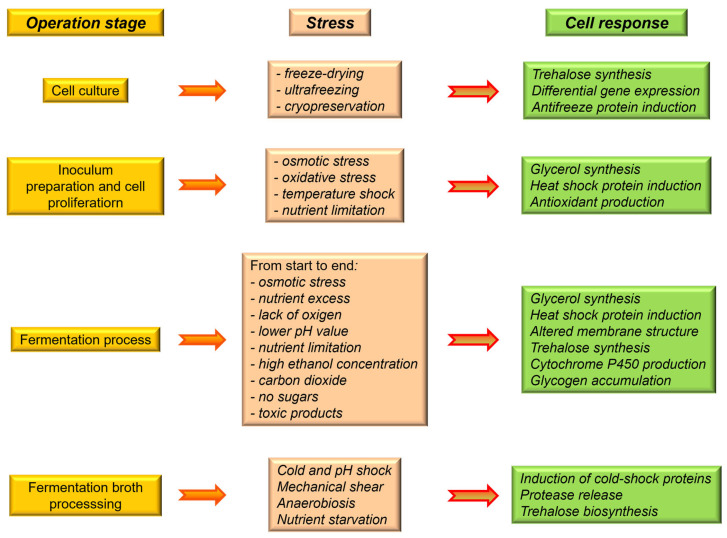
Types of stresses to which the yeast cell can be exposed during the fermentation process and its way of responding to stressful stimuli [33].

With the start of the fermentation process, different stressful factors appear in the environment that directly affect the yeasts. Among them are osmotic, oxidative, and ethanol stresses, nitrogen starvation, low external pH, heat shock, prolonged anaerobiosis or the appearance of toxic molecules, as presented in Figure 1 [33]. As a shield against them, microorganisms have created defense responses specific to each type of stress, as well as a general environmental stress response (ESR) [34,35,36,37].

### 3.1. Oxidative Stress

**Figure 2:**
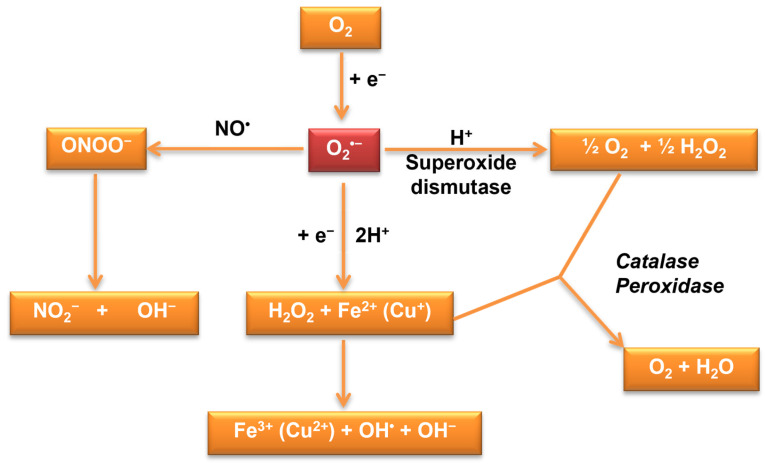
Reactive oxygen species production—antioxidant defense system regulating ROS levels to maintain physiological homeostasis [32].

Oxidative stress results from the cells’ inability to reduce or eliminate reactive oxygen species and reactive nitrogen species (superoxide radical anion O_2_^•−^—the primordial reactive oxygen species, hydroxyl radicals, peroxynitrite, singlet oxygen, and hydrogen peroxide) or to repair the molecular damage produced by them [32,38,39,40,41]. In aerobic conditions, the generation of ROS takes place continuously (Figure 2), these being metabolic side products or the result of cellular control systems. Maintaining low concentrations of reactive oxygen species (ROS), below 10^−8^ M, is achieved by the generation and degradation mechanisms achieved through specific and non-specific cellular mechanisms [42].

Among the factors which determine oxidative stress, metabolism and aerobic respiration contribute the most to this process. A very recent study compared, by using an AI model and neural network algorithm, cellular morphology under basal and stress conditions and concluded that the changes induced by stress due to a high concentration of glucose in the medium were actually due to osmotic stress [43].

In the process of cell development, the microorganism culture goes through several stages, including the lag phase (adaptation), the exponential phase (active cell division), the stationary phase (in which the number of newly formed cells is equal to that of dead cells), and the phase of cell death. In the exponential phase of cell growth, the energy produced is the result of glycolysis. The number of mitochondria is reduced, oxygen consumption is minimal, and so, in this phase, the activity of antioxidant enzymes is also reduced. A responsible factor for oxidative stress is ethanol, by acting on the reactive oxygen species present in the mitochondria [37,44]. In the stationary phase, the cells use the ethanol obtained in the previous phase as an energy source. The number of mitochondria is increased, as a result of their need to oxidize ethanol, so, in this phase, the generation of reactive oxygen species intensifies. Thus, the transition of the cell culture to the stationary phase can cause the occurrence of oxidative stress [44]. The transformation of molecular oxygen into ROS leads to certain phenomena, such as protein oxidation, lipid peroxidation or DNA mutations. These actions occur even though the cells contain antioxidant defense mechanisms [29].

Oxidative stress can also be caused by compounds or substances such as tetrachlorobisphenol A or xylene [45,46]. In addition, through the autooxidation processes of some molecules, some oxidases generate reduced amounts of ROS, such as NADPH oxidase, xanthine oxidase, cyclooxygenases, and lipoxygenases [47].

The defense against oxidative stress is based on enzymatic and non-enzymatic mechanisms, which have the role of maintaining the level of reactive oxygen species within normal limits. The action of the enzymatic part in yeasts is based on the presence of two catalases (A and T) and two superoxide dismutases (Cu/Zn SOD and Mn/Zn SOD). Catalases have the role of breaking down H_2_O_2_ into H_2_O inside the peroxisome and cytosol. Superoxide dismutases convert the superoxide anion into oxygen and H_2_O_2_ in the cytoplasm and mitochondria. Enzymes that act against oxidative stress also play a very important role in the ethanol tolerance of yeasts. They eliminate the reactive oxygen species that appear in the presence of alcohol [37]. Studies have shown that *S. cerevisiae* develops the ability to survive in stress conditions that could become lethal, if it was previously exposed to low doses of cellular stress of the same or a different type [48].

Melatonin is a hormone that protects the human body from oxidative stress, but also from that caused by ethanol. It acts as an antioxidant both for *S. cerevisiae* and for non-*Saccharomyces* microorganisms. Directly, melatonin eliminates reactive oxygen species, and indirectly decreases the amount of oxidized glutathione and activates the genes involved in the response to oxidative stress. Given this information, some studies claim that *S. cerevisiae* produces melatonin to defend itself against the oxidative stress caused by the presence of ethanol and its consequences [37,49,50,51].

Other substances with an antioxidant role are carotenoids. In this case, their chemical structure plays an important role. The nature of the terminal group, the substituents they have in the composition or the length of the polyene chromophore are some of the aspects that determine the stopping of reactive oxygen species from producing unwanted effects [52].

### 3.2. Osmotic Stress—Stress Due to Environmental Salts

*S. cerevisiae* is a microorganism with a high sensitivity to environmental salts. High concentrations can limit water activity, thus leading to cell growth problems [53,54]. Over time, cells have developed a tolerance to high salt concentrations, thus being able to adapt to water fluctuations in the environment. At the same time, this tolerance has an important role in the processes of maintaining or restructuring biological, physiological or morphological functions [53,55,56].

Several strains of *S. cerevisiae* were analyzed to study salt resistance. Following exposure to different molar concentrations of K^+^ (from 0.025 M to 2 M), cells were found to lose viability at 1.5 M K^+^. It was also observed that *S. cerevisiae* strains exhibited a decrease in cell density ranging from 37 to 65% compared to the reference cultures, where they all had approximately the same density. Also, although the concentration of K^+^ increased, some strains of *S. cerevisiae* were able to consume all the glucose in the medium and convert ethanol to acetic acid. In addition to K^+^, the microorganisms were also exposed to different concentrations of Na^+^. Some *S. cerevisiae* strains showed a 79% decrease in growth rate at only 0.5 M Na^+^, while others had a 59% decrease at 2 M Na^+^. In the case of sodium use, the cell density of *S. cerevisiae* chains was between 25% and 68% compared to the reference values [53].

Some studies have shown, following the analysis of the microorganism *Deinococcus radiodurans*, that it has a gene which increases its tolerance to environmental stress. This was isolated and then combined with the genetic material of a strand of *S. cerevisiae*. After exposure to different salt concentrations (2%, 3%, 4%, 5%, and 7%), it was observed that normal strands of *S. cerevisiae* can withstand a maximum salt content in the medium of 5%. However, the strand of *S. cerevisiae* that contained the *pprI* gene, originating from the microorganism *Deinococcus radiodurans*, tolerated the salt concentration of 7% very well [16].

Another recent study investigated how the adhesion and cytotoxicity of positively charged polystyrene nanoparticles from two yeast cultures are affected in the presence of the NaCl salt. The nanocarriers were exposed for 24 h to NaCl concentrations ranging from 5 to 600 mM and temperatures from 4 to 25 °C. The yeast strains used were *Saccharomyces cerevisiae* and *Schizosaccharomyces pombe*. At the end of the research, it was observed that, for the survival of the two cultures, at a temperature of 4 °C, NaCl concentrations of 100 mM for *S. pombe* and 150 mM for *S. cerevisiae* were necessary. In the case of *S. cerevisiae*, the degree of adhesion decreased with increasing NaCl concentration at a temperature of 25 °C and an exposure time of less than 4 h. Thus, at NaCl values higher than 150 mM, the adhesion became almost insignificant. In terms of cytotoxicity, the higher the degree of adhesion at the beginning, the more pronounced the cell death. Regarding *S. pombe*, the degree of adhesion increased at the same time as the NaCl concentration. Thus, it was observed that the cells coated with nanoparticles had a mortality reduced by 50% at an exposure time of less than 4 h [57].

Water activity describes the chemical yield of free water in a solution. One of its uses is to help classify microorganisms into: halophile, halotolerant, and halosensitive. Halophile microorganisms have the ability to grow in environments with high salinity, while halo-sensitive ones are drastically affected by such amounts. At their core, halo-tolerant microorganisms are those that do not require certain amounts of salt to grow and can be viable over a wide range of salinity values in the environment. In order to be classified into one of the categories above, yeasts must have a certain value for water activity. Thus, for a value lower than 0.70, microorganisms are considered halophile. However, most of them develop at values between 0.90 and 0.95 [58]. Some examples of halo-tolerant yeasts are described in Table 1.

Stress caused by environmental salts can lead to hyperosmotic stress, but also to specific cation toxicity. Its action on *S. cerevisiae* causes the microorganism to activate certain protective steps. First, some mechanisms are activated that prevent cell death following the change in osmolarity. Processes ensue that are intended to protect, repair, and recover from the osmotic effects and sodium toxicity. Finally, cells resume their growth process through readaptation conditions. However, *S. cerevisiae* has a long-term defense process that relies on osmotic changes driven by osmolyte synthesis and cation transport to remove sodium [59].

The waste water produced in the pharmaceutical, textile, chemical or food industries is very harmful to the environment. It contains large amounts of pollutants and salts that make the biodegradation process difficult. Although there are certain treatment processes, they involve the appearance of new substances at the end. For this reason, bioaugmentation was researched. The technology accelerates the destruction of refractory pollutants by involving microorganisms in a classic biological treatment system. Thus, it was found that halophile yeasts are effective in cleaning polluted waters. An example is *Meyerozyma guilliermondii* W2, which has a high degree of survivability and growth in high salinity environments. In one study, the use of microorganisms increased the chemical oxygen demand (COD) removal percentage from 67% to 94% after the bioaugmentation process [60].

Related to the salt tolerance of yeasts, a study was carried out with the aim to determine the effects of carotenoid substances in the defense against salt stress. Thus, strains of *Sporidiobolus pararoseus* NGR were exposed to different concentrations of NaCl. HPLC results showed that carotenoid products released by *Sporidiobolus pararoseus* NGR protect microorganisms against salt stress. Separately, amounts of diphenylamine were added to some samples to evaluate the behavior of carotenoids. It was observed that the synthesis of the substances was inhibited, thus no longer able to protect the cells of *Sporidiobolus pararoseus* NGR against NaCl in the environment [52].

### 3.3. Stress Due to Ethanol Accumulation

Ethanol is both the product resulting from the alcoholic fermentation of yeasts, but also one of the factors that lead to the appearance of stress at the cellular level. As the fermentation process progresses, more and more ethanol is produced. This leads to harmful actions against the yeast cells, such as blocking cell proliferation, depolarizing the cytoskeleton, or altering the activity of transport systems [61]. To respond effectively to stress conditions, yeast reprograms its cellular activity to protect important cell components for normal cellular activity and a higher degree of survival [62].

The structure of the yeast cell wall protects it against permanent changes in the external environment and it prevents cell lysis resulting from changes in osmotic conditions. The alcohol causes the disruption of cell integrity by intercalation in the hydrophilic layer inside the lipid bilayer of the membrane, thus increasing cell permeability [48,63]. The presence of ethanol during fermentation subjects yeasts to continuous stress. Thus, most of the time, cell viability is reduced, followed by the full blockage of the process for obtaining a certain product [37]. To prevent this, cells use several protective mechanisms. Among them are the activation of some heat shock proteins, the increase in the level of unsaturated fatty acids and ergosterol in the plasma membrane or the accumulation of the intracellular trehalose (known to replace the water molecule to stabilize proteins and membranes from desiccation and to protect yeast cells from thermal denaturation) content in the cells [64,65,66,67]. Additionally, studies demonstrate that ATPase in the plasma membrane also controls the tolerance of yeast cells to the stress due to ethanol, as this enzyme is reported to be activated by the alcohol [68]. Vacuolar and membrane ATPase are involved in the recovery after the acidification at a cytosol level induced by the presence of alcohol, by pumping protons into the vacuole and outside the cell, due the effect of alcohol in order to induce a membrane permeabilization effect through a passive influx of protons [62,69,70].

As for the concentration of ethanol in the environment, it produces certain effects both at low and high levels. At a low percentage, the yeast cells undergo a slowdown in division, resulting in slower growth. At high concentration, the fluidity of the cell membrane is improved, but cell viability decreases, the electrochemical gradient decreases, and the activity of glycolytic enzymes is affected by protein denaturation and the increase in the percentage of insoluble proteins [67]. By analyzing the effect of ethanol concentration on yeast proteins, it was observed that the inhibition of the process is dependent on it. To support the statement, a study was carried out on two cultures of *Saccharomyces cerevisiae*, one commercial, for wine production, and one of laboratory type. Both were subjected to alcohol concentrations from 6% to 14%. Cell growth was completely stopped at a concentration of 12% for laboratory strain and 14% for the commercial one. In another study, one set of cells was subjected to 10% ethanol. As a result, protein aggregates formed and the translation of the genetic material was repressed. Thus, it can be said that, at values higher than 10%, the degradation of the proteasome is strongly affected [37,61].

The ethanol resulting from fermentation has harmful effects on the yeast cell membrane. To avoid cell damage, some studies have shown that Mg^2+^ ions exhibit protective actions against ethanol stress. Other researchers report that maintaining a balance of K^+^ ions in the cell membrane lessens ethanol stress, increasing fermentative activity. Resveratrol is a polyphenol capable of increasing ethanol tolerance. This increase occurs by decreasing lipid peroxidation and superoxide dismutase activity [2,37].

Sometimes, the exposure of yeast cells to ethanol stress may have positive effects. For example, the activity of the ubiquitin–proteasome proteolytic system can be analyzed during this process. For certain proteins, ethanol leads to the suppression of proteolysis, but after its removal, the degradation resumes. In other words, the inhibition of proteasomal degradation is a reversible process [61].

## 4. The Behavior of *Saccharomyces cerevisiae* in Stressors Action

Once environmental stress acts on microorganisms, it produces changes at a genetic and molecular level. For example, yeast cells produce much higher amounts of trehalose in response to heat stress and the disaccharide acts to stabilize plasma membranes. Also, some genes that have a role in the synthesis of ergosterols are able to exert thermotolerance properties in yeasts [2].

**Figure 3:**
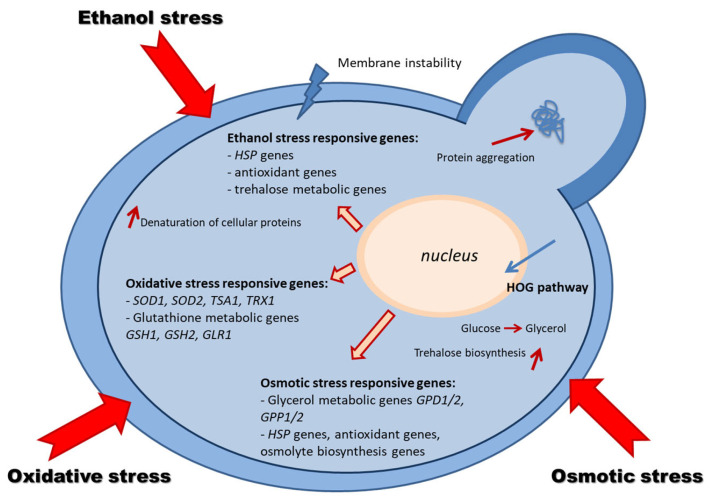
Molecular mechanisms of osmotic, ethanol, and oxidative stress response in yeast [62,70].

An overview of the yeast adaptive response in osmotic, ethanol, and oxidative stress is presented in Figure 3 [62,70].

Yeast cells adapt to stress by activating or inhibiting the genes responsible for actions against it. An alternative is epigenetic mechanisms by which yeasts quickly adapt to cellular stress. These processes involve an organization of chromatin into histones, DNA modifications or changes in transcription patterns [13,71,72,73]. Certain responses of yeast cells to environmental stress are shown in Table 2.

Due to problems with nutrient-poor environments or poor parameters control during fermentation, the conversion of sugar to alcohol is often slowed down. Apart from these causes, there are also certain factors that cause damage during the process of obtaining alcohol. Osmotic stress, ethanol toxicity or certain pH and temperature values can affect the growth of yeast cells, but also their metabolism. For example, an ethanol concentration greater than 10% (*v*/*v*) and a temperature above 35 °C greatly reduce the viability of microorganisms [2].

Zinc is an important micronutrient in the growth process of *S. cerevisiae* cells. About 3% of their proteome requires zinc to function properly. In other words, 105 proteins use it as a cofactor, and another 360 need it to maintain structural stability through binding domains where it is a key factor. The trace element also enters into the structure of some proteins called zinc fingers, these being the largest family of proteins that bind nucleic acids and that have an important function in regulating transcription [74]. Table 3 lists some examples and how they act against cellular stress in the yeast *S. cerevisiae*.

Different yeasts from the class *Saccharomycetes* have been analyzed and it has been observed that each exhibits distinct defense techniques against osmotic and salt stress. For example, *Zygosaccharomyces rouxii* and *S. cerevisiae* can export Na^+^ cations out of the cell or into vacuoles. In contrast, *Debaryomyces hausenii* accumulates Na^+^ ions in the cell without suffering intoxication [75]. Table 4 highlights the cation transport systems in several yeast species. For yeast species where a gene is present, a “+” was marked in the corresponding box. In the opposite sense, a “−” was entered.

Thanks to the success of sequencing the S288c strain from *S. cerevisiae*, a coordination system between the extracellular environment and the changes occurring in the cell could be created. The mechanism is based on receiving information from the environment, transmitting it internally, and adapting it with the genetic information of the cell to create an appropriate response. The system was later used in the yeast fermentation process to optimize it at high salt concentrations [54,76].

In one study, *S. cerevisiae* cells were subjected to a pulsed electric field to observe its effect on them. In addition, they were also exposed to heat and the first strand of the complementary DNA obtained from the RNA of the cells was analyzed. The genes of interest were those responding to oxidative and thermal stress. Heat stress led to the activation of the *MSP104* gene, which encodes the heat shock protein. In contrast, the expression of the oxidative stress response gene *GLR1* was inhibited. On the other hand, the pulsed electric field exhibited the opposite, stimulating the *GLR1* gene and repressing *MSP104* [77].

During an industrial process, it is important that the chosen microorganism has increased tolerance to the stress that may occur after a certain period of time. Thus, specialists resort to physiological and genetic methods to improve the resistance of the chains of *Saccharomyces* used. One of them refers to adaptive evolution, which involves a gradual and prolonged exposure to certain values of environmental factors. In this way, the microorganism develops a higher capacity to face the difficult conditions that may occur until the end of fermentation [2]. Table 5 mentions some actions that can be carried out to reduce the stress that occurs during fermentation.

The genetic modifications made to improve the chains of *S. cerevisiae* also play an important role. Mutagenesis, hybridization, and protoplast fusion are some of the classic techniques used to increase stress resistance. As for newer methods, gene editing technologies show promising results in this area. With the help of one of these, the desired genes can be deleted and inserted as precisely as possible by clustered and regularly interspersed palindromic repeats (CRISPR) and by CRISPR nuclease associated with protein 9 (Cas9). Following application, tolerance to ethanol, acetic acid, and temperature were increased considerably. In the culture medium, stress can occur by itself under the action of certain factors, but it can also be induced to manipulate the cells to produce as much of the final product as possible. Table 6 shows some examples of how types of stress are used to influence yeasts [2].

Some of the common genes responsible for yeast cells’ adaptation to different stresses are presented in Table 7 [78,79].

In order to adapt to the conditions to which they are exposed, especially to stress, yeast cells go through certain changes at the proteome level [35,80]. A set of proteins that produce positive effects on *S. cerevisiae* is represented by Msn2 and Msn4 (Msn 2/4). They are able to stop the mutation of the *SNF1* gene and regulate some stress responses. Through cycles of phosphorylation and dephosphorylation, proteins are activated to produce certain effects. For example, ethanol tolerance and fermentation improvement in the yeast *S. cerevisiae* can be influenced after the phosphorylation of specific serine residues of Msn 2/4. Of course, proteins can also act in a less desirable way on *S. cerevisiae*. They can decrease their growth rate by overwriting the binding domain of their DNA [81]. The phosphorylation processes controlling Msn2/4 are represented by low protein kinase A (PKA) activity and low nitrogen and glucose concentrations. Instead, dephosphorylation relies on intense PKA activity and high glucose concentration. In addition to these two, the functions of Msn2/4 are also influenced by the nuclear and cytoplasmic localization of the reactions taking place under the catalysis of the protein kinase Tpk1-3, specific for the cAMP-PKA signaling pathway. In other words, it can be said that this pathway is responsible for the thermotolerance of *S. cerevisiae* cells, the influence of some of the cellular processes and the accumulation of stress-resistant substances (glycerol, trehalose, and glycogen). Also, both under normal conditions and under cellular stress, the cAMP-PKA pathway can identify and respond to high or low amounts of nitrogen and glucose [64,82]. In the case of the yeast *S. cerevisiae*, the cAMP-PKA signaling pathways contain elements such as Msn2/4 (transcription activating proteins), Bcy1-2 (regulatory subunit of PKA), Mep2 (ammonium permease/nitrogen sensor) or Grl1 (sensing glucose, activator of adenylate cyclase). In cAMP-PKA signaling, the DNA binding domain of Msn2/4 has the ability to recognize promoter sequences (AG4 and C4T). At high glucose concentrations, Bcy1 is removed from the catalytic subunits Tpk1-3, and thus, an increase in Tpk1-3 activity results. At the same time, Msn2/4 phosphorylation occurs, followed by expulsion from the cell nucleus [82].

Another signaling pathway present in *S. cerevisiae* cells is that of nitrogen or TORC1 (target of rapamycin 1). It includes Sch9 (protein kinase), TORC1 (is the key regulator, stimulated by nitrogen, activates Sch9 by phosphorylation), Deh1, and Dal80 (represses transcription) and Gat1, Glu3, and Msn 2/4 (activates transcription). The role of the TORC1 signaling pathway is to phosphorylate Glu3, Gat1, and Msn 2/4 and to inhibit the phosphorylation of Deh1 and Dal80. These actions only occur in the presence of high concentrations of nitrogen and amino acids. As long as the nitrogen signaling pathway is active, the expression of the genes responsible for inducing cellular stress is stopped [83,84].

## 5. Conclusions

In the fermentation process, yeast cells are continuously and simultaneously subjected to different types of cellular stress, which determines a constant cellular adaptation to environmental conditions. This paper focuses on the specific response to each type of stress that occurs in the cell—osmotic, oxidative or due to the presence of ethanol—by means of the arduous biochemical and transcription regulation or stimulation of certain genes processes. Under the action of various stressors, yeast cells can modify their colony growth morphological architecture and changes occur at a genetic level. The response to cellular stress involves the action of multiple genes, such as *SOD1*, *SOD2*, *TSA1*, *GSH1*, *GSH2*, *GLR1*, and *CTT1* (oxidative stress), *GPD1*, *GPD2*, and *HSP12* (osmotic stress), and *HSP104*, *GUP1*, *GPP1*, *GPP2*, *GPD1*, *GAT1*, and *OLE1* (ethanol stress). Further molecular biology and genetics studies are necessary for the complete understanding of the cellular regulation mechanisms under stress conditions. This information could lead to the optimal use of *Saccharomyces cerevisiae* species in industrial fermentation processes and to an increase in the bioproducts’ quality. In addition, deciphering the physiological and genetic response of stress mitigation in yeast cells can also lead to countering the aging effects on human cells.

## Figures and Tables

**Table 1:** Halotolerant yeast species [54].

Category	Characteristic	Species Name	NaCl: M; % (*w*/*v*)
Halotolerant	Growth at NaCl > 2.0 M	*C. parapsilosis*	3.0; 17.5
		*P. membranifaciens*	3.0; 17.5
		*I. orientalis*	2.0; 11.7
		*P. sorbitophila*	3.0–4.0; 17.5–23.4
		*Z. rouxii*	3.0; 17.5
		*H. werneckii*	5.20; 30.8
		*D. hansenii*	3.0–4.0; 17.5–23.4
		*C. halophila*	4.0–5.0; 23.4–29.1
Moderate halotolerant	Growth inhibition at NaCl > 2.0 M	*S. cerevisiae*	<1.70; 10
		*S. pombe*	1.00; 5.8
		*Z. florentinus*	1.00; 5.8
		*C. albicans*	1.70; 10
		*C. tropicalis*	1.7–2.0; 10–11.7
		*Z. sapae*	2.0; 11.7
		*Z. bailii*	1.0–2.0; 5.8–11.7
		*Z. bisporus*	1.0–2.0; 5.8–11.7

**Table 2:** *S. cerevisiae* stress response [2].

Stress Response	Details
Apoptosis	Near the end of life, senescent cells enter apoptosis
Genotypic changes	Mutations are common in yeasts subjected to stress during the brewing process
Changes in intracellular metal ion homeostasis	Several stressors lead to the loss of key metal ions such as Mg and Zn
Blocking the cell division cycle	Stress leads to the blocking of the yeast cell cycle, thus inhibiting growth
Induction of stress enzymes	For example, the induction of antioxidant enzymes during the action of oxidative stress
Activation of glycerol biosynthesis	Glycerol is produced in excess during osmotic stress
Induction of trehalose biosynthesis	Cells produce large amounts of trehalose in response to heat shock and other stressors
Structural changes in the cell membrane	Disorders of membrane integrity caused by stressors
Induction of heat shock protein synthesis (hot/cold)	Heat shock proteins are induced in response to high temperatures and other factors

**Table 3:** Zinc finger proteins with role in stress tolerance of *S. cerevisiae* yeast [74].

Gene Type	Function
*Ace2*	Transcription factor that early activates G1-specific gene expression; may be involved in acetic acid tolerance
*Crz1*	Transcription factor that activates the copying of genes involved in the stress response
*Nrg1*	Intervenes in glucose repression and negatively regulates many processes, including filamentous growth. Involved in tolerance to salt, oxidative stress, and acetic acid
*Rim101*	Repressive transcription factor involved in cell wall structuring and pH response. It also plays a role in acetic acid tolerance
*Stb5*	Transcription factor involved in multidrug resistance, oxidative stress response, and acetic acid tolerance
*Usv1*	Transcriptional regulator of genes involved in growth of non-fermentable carbon sources and response to osmotic stress
*Cmr3*	YPR015C-related transcription factor affects the copying of genes involved in cell defense and rescue, but also in DNA processing and the cell cycle
*Msn2/4*	Activators of transcription, regulate the general stress response. Overwriting Msn2 improves ethanol tolerance
*Prd1/Prd3*	Involved in tolerance to hydroxymethylfurfural (HMF) and ethanol, and in response to salt stress
*Ume6*	Key transcriptional regulator of early meiotic genes, involved in acetic acid tolerance and salt stress response

**Table 4:** Gene encoding cation transport associated with salt tolerance in different yeast species [54,75].

System for	Gene	*Sc* ^1^	*Sp* ^2^	*Zr* ^3^	*Dh* ^4^	*Ca* ^5^
K^+^ influx	*TRK1*	+	+	+	+	+
	*TRK2*	+	+	−	−	−
	*HAK1*	−	−	−	+	+
	*ACU1*	−	−	−	−	pseudogene
K^+^ efflux	*TOK1*	+	−	+	−	+
K^+^ and Na^+^ efflux	*NHA1*	+	+	+	+	+
	*SOD2*	−	+	+	undefined	−
	*ENA1-4*	+	+	+	+	+

^1^ S. cerevisiae, ^2^ S. pombe, ^3^ Z. rouxii, ^4^ D. hansenii, ^5^ C. albicans.

**Table 5:** Ways to reduce stress on yeast cells [2].

Method	Details
Microbial contamination control	Sanitization operations of fermentation processes, use of antimicrobial products
Ensuring optimal physiological conditions	Correct cold storage temperature, optimal pre-propagation conditions
Use of improved yeast strains	Application of genetic and physiological techniques to optimize stress resistance
Ensuring proper yeast nutrition	Micronutrient balance, bioavailability for metal ions
Control of fermentation parameters	pH, temperature, agitation, specific density of the medium, dissolved oxygen

**Table 6:** Influence of stress on yeasts in industrial applications [2].

Stress Type	Cell Response	Industrial Application
Osmotic shock	Glycerol and trehalose levels increase by lowering the osmotic potential of the growth medium	Usage of tolerant yeasts in fermentation processes
UV irradiation	Irradiated yeasts convert membrane ergosterol to vitamin D2	Vitamin-D2-enriched yeasts for baking and nutraceuticals
Oxidative stress	Induction of catalase and superoxide dismutase, stimulation of membrane sterol, and unsaturated fatty acid synthesis in aerobic environment	Oxygenated yeasts will have a higher stress tolerance during the fermentation process due to the high amounts of ergosterol and oleic acid in the membrane
Heat shock	Yeast cells with high levels of trehalose have a higher tolerance against stress after heat shock	Usage of frozen dough for baking
Heat and salts	Self-digestion	Obtaining yeast extracts and yeast beta-glucan

**Table 7:** *Saccharomyces cerevisiae* common genes with a role in stress response [78,79].

Cell Stress Genes	Biological Stress Response
*BSD2*	involved in metal ion transport (IMP), protein targeting to vacuole (IMP), ubiquitin-dependent protein catabolic process (IMP)
*CTT1*	involved in response to reactive oxygen species (IMP), protection from oxidative damage by hydrogen peroxide
*DAK1*	involved in glycerol to glycerone phosphate metabolic process (IGI), involved in stress adaptation
*DDR48*	involved in DNA repair (IDA, IMP), expression is increased in response to heat-shock stress
*GTT1*	involved in glutathione metabolic process (IDA) and protein glutathionylation (IMP)
*HSP104*	involved in cellular heat acclimation (IMP), chaperone cofactor-dependent protein refolding (IDA), protein folding in endoplasmic reticulum (IMP), protein unfolding (IMP), trehalose metabolism in response to heat stress (IMP), responds to stresses including heat, ethanol, and sodium arsenite
*HSP12*	involved in cell adhesion (IDA), in plasma membrane organization (IMP), cellular response to heat (IMP), osmotic stress (IMP), and oxidative stress (IMP)
*HSP26*	involved in cellular response to heat (IDA) and in protein folding (IDA)
*HSP42*	involved in cytoskeleton organization after heat shock (IMP)
*HSP78*	involved in cellular response to heat (IMP, IGI), mitochondrial genome maintenance (IGI), protein refolding (IDA, IMP), stabilization (IMP, IGI), and unfolding (IMP)
*NCE103*	involved in cellular response to carbon dioxide (IMP) and oxidative stress (IMP)
*PPZ2*	involved in intracellular sodium ion homeostasis (IMP) and regulation of potassium transport, which affects osmotic stability
*SSA4*	involved in protein folding (IGI), cellular response to heat, and role in SRP-dependent cotranslational protein-membrane targeting to membrane and translocation (IMP)
*TRR2*	involved in protection against oxidative stress (IMP)
*TTR1*	involved in cellular response to oxidative stress (IMP) and in glutathione metabolic process (IGI)
*XBP1*	involved in negative regulation of transcription by RNA polymerase II (IMP), induced by stress or starvation during mitosis, and late in meiosis
*TSA2*	involved in cell redox homeostasis (IDA, IMP), in cellular response to oxidative stress (IGI, IMP), and protein folding (IDA)
*NQM1*	involved in cellular response to oxidative stress (IMP)
*RCN1*	involved in response to osmotic and ionic changes concentration

IMP—Inferred from Mutant Phenotype, IGI—Inferred from Genetic Interaction, IDA—Inferred from Direct Assay.

## References

[1] Costa A.C.T., Russo M., Fernandes A.A.R., Broach J.R., Fernandes P.M.B. (2023). Transcriptional Response of Multi-Stress-Tolerant *Saccharomyces cerevisiae* to Sequential Stresses. Fermentation.

[2] Walker G.M., Basso T.O. (2020). Mitigating stress in industrial yeasts. Fungal Biol..

[3] Kyriakou M., Christodoulou M., Ioannou A., Fotopoulos V., Koutinas M. (2023). Improvement of stress multi-tolerance and bioethanol production by *Saccharomyces cerevisiae* immobilised on biochar: Monitoring transcription from defence-related genes. Biochem. Eng. J..

[4] Gibson B.R., Lawrence S.J., Leclaire J.P.R., Powell C.D., Smart K.A. (2007). Yeast response to stresses associated with industrial brewery handling. FEMS Microbiol. Rev..

[5] Cavalieri D., McGovern P.E., Hartl D.L., Mortimer R., Polsinelli M. (2003). Evidence for *S. cerevisiae* Fermentation in Ancient Wine. J. Mol. Evol..

[6] McGovern P.E., Zhang J., Tang J., Zhang Z., Hall G.R., Moreau R.A., Nunez A., Butrym E.D., Richards M.P., Wang C. (2004). Fermented beverages of pre- and proto-historic China. Proc. Natl. Acad. Sci. USA.

[7] De Llanos R., Llopis S., Molero G., Querol A., Gil C., Fernandez-Espinar M.T. (2011). In vivo virulence of commercial *Saccharomyces cerevisiae* strains with pathogenicity-associated phenotypical traits. Int. J. Food Microbiol..

[8] Pillai U., Devasahayam J., Kurup A.N., Lacasse A. (2014). Invasive *Saccharomyces cerevisiae* Infection: A Friend Turning Foe?. Saudi J. Kidney Dis. Transpl..

[9] Cui L., Lucht L., Tipton L., Rogers M.B., Fitch A., Kessinger C., Camp D., Kingsley L., Leo N., Greenblatt R.M. (2015). Topographic Diversity of the Respiratory Tract Mycobiome and Alteration in HIV and Lung Disease. Am. J. Respir. Crit. Care Med..

[10] Zhu Y.O., Sherlock G., Petrov D.A. (2016). Whole Genome Analysis of 132 Clinical *Saccharomyces cerevisiae* Strains Reveals Extensive Ploidy Variation. G3.

[11] Ackerman A.L., Underhill D.M. (2017). The mycobiome of the human urinary tract: Potential roles for fungi in urology. Ann. Transl. Med..

[12] Nash A.K., Auchtung T.A., Wong M.C., Smith D.P., Gesell J.R., Ross M.C., Stewart C.J., Metcalf G.A., Muzny D.M., Gibbs R.A. (2017). The gut mycobiome of the Human Microbiome Project healthy cohort. Microbiome.

[13] Raghavan V., Aquadro C.F., Alani E. (2019). Baker’s Yeast Clinical Isolates Provide a Model for How Pathogenic Yeasts Adapt to Stress. Trends Genet..

[14] Kitagaki H., Takagi H. (2014). Mitochondrial metabolism and stress response of yeast: Applications in fermentation technologies. J. Biosci. Bioeng..

[15] Zhao X.Q., Bai F.W. (2009). Mechanisms of yeast stress tolerance and its manipulation for efficient fuel ethanol production. J. Biotechnol..

[16] Helalat S.H., Bidaj S., Samani S., Moradi M. (2019). Producing alcohol and salt stress tolerant strain of *Saccharomyces cerevisiae* by heterologous expression of pprI gene. Enzyme Microb. Technol..

[17] Steen E.J., Chan R., Prasad N., Myers S., Petzold C.J., Redding A., Ouellet M., Keasling J.D. (2008). Metabolic engineering of *Saccharomyces cerevisiae* for the production of n-butanol. Microb. Cell Fact..

[18] Goffeau A., Barrell B.G., Bussey H., Davis R.W., Dujon B., Feldmann H., Galibert F., Hoheisel J.D., Jacq C., Johnson M. (1996). Life with 6000 genes. Science.

[19] Barnett J.A. (2003). Beginnings of microbiology and biochemistry: The contribution of yeast research. Microbiology.

[20] Cherry J.M., Hong E.L., Amundsen C., Balakrishnan R., Binkley G., Chan E.T., Christie K.R., Costanzo M.C., Dwight S.S., Engel S.R. (2012). *Saccharomyces* Genome Database: The genomics resource of budding yeast. Nucleic Acids Res..

[21] Hagman A., Säll T., Compagno C., Piskur J. (2013). Yeast “Make-Accumulate-Consume” Life Strategy Evolved as a Multi-Step Process That Predates the Whole Genome Duplication. PLoS ONE.

[22] Engel S.R., Dietrich F.S., Fisk D.G., Binkley G., Balakrishnan R., Costanzo M.C., Dwight S.S., Hitz B.C., Karra K., Nash R.S. (2014). The reference genome sequence of *Saccharomyces cerevisiae*: Then and now. G3.

[23] Williams K.M., Liu P., Fay J.C. (2015). Evolution of ecological dominance of yeast species in high-sugar environments. Evolution.

[24] Basile A., De Pascale F., Bianca F., Rossi A., Frizzarin M., De Bernardini N., Bosaro M., Baldisseri A., Antoniali P., Lopreiato R. (2021). Large-scale sequencing and comparative analysis of oenological *Saccharomyces cerevisiae* strains supported by nanopore refinement of key genomes. Food Microbiol..

[25] Saito T.L., Ohtani M., Sawai H., Sano F., Saka A., Watanabe D., Yukawa M., Ohya Y., Morishita S. (2004). SCMD: *Saccharomyces cerevisiae* Morphological Database. Nucleic Acids Res..

[26] Stewart G.G., Batt C.A., Tortorello M.L. (2014). SACCHAROMYCES|Saccharomyces cerevisiae. Encyclopedia of Food Microbiology.

[27] Granek J.A., Magwene P.M. (2010). Environmental and Genetic Determinants of Colony Morphology in Yeast. PLoS Genet..

[28] Kaeberlein M., Burtner C.R., Kennedy B.K. (2007). Recent developments in yeast aging. PLoS Genet..

[29] Inderpal M. (2017). Oxidative Stress in *Saccharomyces cerevisiae*. Doctoral Dissertation.

[30] Cazzanelli G., Pereira F., Alves S., Francisco R., Azevedo L., Carvalho P.D., Almeida A., Corte-Real M., Oliveira M.J., Lucas C. (2018). The Yeast *Saccharomyces cerevisiae* as a Model for Understanding RAS Proteins and Their Role in Human Tumorigenesis. Cells.

[31] Nielsen J. (2019). Yeast Systems Biology: Model Organism and Cell Factory. Biotechnol. J..

[32] Eleutherio E., Brasil A.A., França M.B., de Almeida D.S.G., Rona G.B., Magalhães S.S. (2018). Oxidative stress and aging: Learning from yeast lessons. Fungal Biol..

[33] Walker G.M., Van Dijck P., Querol A., Fleet G.H. (2006). Physiological and Molecular Responses of Yeasts to the Environment. Yeasts in Food and Beverages.

[34] Penninckx M. (2000). A short review on the role of glutathione in the response of yeasts to nutritional, environmental, and oxidative stresses. Enzyme Microb. Technol..

[35] Crawford R.A., Pavitt G.D. (2019). Translational regulation in response to stress in *Saccharomyces cerevisiae*. Yeast.

[36] Eardley J., Timson D.J. (2020). Yeast Cellular Stress: Impacts on Bioethanol Production. Fermentation.

[37] Sunyer-Figueres M., Mas A., Beltran G., Torija M.J. (2021). Protective Effects of Melatonin on *Saccharomyces cerevisiae* under Ethanol Stress. Antioxidants.

[38] Herrero E., Ros J., Belli G., Cabiscol E. (2008). Redox control and oxidative stress in yeast cells. Biochim. Biophys. Acta.

[39] Moreno-Garcia J., Mauricio J.C., Moreno J., Garcia-Martinez T. (2016). Stress responsive proteins of a flor yeast strain during the early stages of biofilm formation. Process Biochem..

[40] Vázquez J., Grillitsch K., Daum G., Mas A., Beltran G., Torija M.J. (2019). The role of the membrane lipid composition in the oxidative stress tolerance of different wine yeasts. Food Microbiol..

[41] Ayer A., Fazakerley D.J., James D.E., Stocker R. (2022). The role of mitochondrial reactive oxygen species in insulin resistance. Free Radic. Biol. Med..

[42] Lushchak V.I. (2011). Adaptive response to oxidative stress: Bacteria, fungi, plants and animals. Comp. Biochem. Physiol. C Toxicol. Pharmacol..

[43] Itto-Nakama K., Watanabe S., Ohnuki S., Kondo N., Kikuchi R., Nakamura T., Ogasawara W., Kasahara K., Ohya Y. (2023). Prediction of ethanol fermentation under stressed conditions using yeast morphological data. J. Biosci. Bioeng..

[44] Lushchak V.I. (2010). Oxidative stress in yeast. Biochemistry.

[45] Ji Z., Zhang Y., Tian J., Wang F., Song M., Li H. (2018). Oxidative stress and cytotoxicity induced by tetrachlorobisphenol A in *Saccharomyces cerevisiae* cells. Ecotoxicol. Environ. Saf..

[46] Yashimoto N., Kawai T., Yoshida M., Izawa S. (2019). Xylene causes oxidative stress and pronounced translation repression in *Saccharomyces cerevisiae*. J. Biosci. Bioeng..

[47] Puddu P., Puddu G.M., Cravero E., Rosati M., Muscari A. (2008). The molecular sources of reactive oxygen species in hypertension. Blood Press..

[48] Ndukwe J.K., Aliyu G.O., Onwosi C., Chukwu K.O., Ezugworie F.N. (2020). Mechanisms of weak acid-induced stress tolerance in yeasts: Prospects for improved bioethanol production form lignocellulosic biomass. Process Biochem..

[49] Romero A., Ramos E., de Los Rios C., Egea J., del Pino J., Reiter R.J. (2014). A review of metal-catalyzed molecular damage: Protection by melatonin. J. Pineal Res..

[50] Bisquert R., Muniz-Calvo S., Guillamon J.M. (2018). Protective Role of Intracellular Melatonin Against Oxidative Stress and UV Radiation in *Saccharomyces cerevisiae*. Front. Microbiol..

[51] Morcillo-Parra M.A., Beltran G., Mas A., Torija M.J. (2020). Effect of Several Nutrients and Environmental Conditions on Intracellular Melatonin Synthesis in *Saccharomyces cerevisiae*. Microorganims.

[52] Li C., Zhang N., Li B., Xu Q., Song J., Wei N., Wang W., Zou H. (2017). Increased torulene accumulation in red yeast *Sporidiobolus pararoseus* NGR as stress response to high salt conditions. Food Chem..

[53] Illarionov A., Lahtvee P.-J., Kumar R. (2021). Potassium and Sodium Salt Stress Characterization in the Yeasts *Saccharomyces cerevisiae*, *Kluyveromyces marxianus*, and *Rhodotorula toruloides*. Appl. Environ. Microbiol..

[54] Dakal T.C., Solieri L., Giudici P. (2014). Adaptive response and tolerance to sugar and salt stress in the food yeast *Zygosaccharomyces rouxii*. Int. J. Food Microbiol..

[55] Yancey P.H. (2005). Organic osmolytes as compatible, metabolic and counteracting cytoprotectants in high osmolarity and other stresses. J. Exp. Biol..

[56] Klipp E., Nordlander B., Kruger R., Gennemark P., Hohmann S. (2005). Integrative model of the response of yeast to osmotic shock. Nat. Biotechnol..

[57] Shinto H., Kojima M., Shigaki C., Hirohashi Y., Seto H. (2022). Effect of salt concentration and exposure temperature on adhesion and cytotoxicity of positively charged nanoparticles toward yeast cells. Adv. Powder Technol..

[58] Tokuoka K. (1993). Sugar- and salt-tolerant yeasts. J. Appl. Bacteriol..

[59] Dinu L.D., Craciun T. (2022). Salt stress resistance in yeast is heterogeneous. Period. Mineral..

[60] Wen H., Xiong K., Yang H., Zhang P., Wang X. (2022). Dynamic mechanism of the microbiota of high-salinity organic wastewater with salt-tolerant yeast and its application. J. Environ. Chem. Eng..

[61] Nguyet V.T.A., Furutani N., Ando R., Izawa S. (2022). Acquired resistance to severe ethanol stress-induced inhibition of proteasomal proteolysis in *Saccharomyces cerevisiae*. Biochim. Biophys. Acta Gen. Subj..

[62] Saini P., Beniwal A., Kokkiligadda A., Vij S. (2018). Response and tolerance of yeast to changing environmental stress during ethanol fermentation. Process Biochem..

[63] Charoenbhakdi S., Dokpikur T., Burphan T., Techo T., Auesukaree C. (2016). Vacuolar H^+^-ATPase protects *Saccharomyces cerevisiae* cells against ethanol-induced oxidative and cell wall stresses. Appl. Environ. Microbiol..

[64] Francois J.M., Walther T., Parrou J.L., Liu Z. (2012). Genetics and Regulation of Glycogen and Trehalose Metabolism in *Saccharomyces cerevisiae*. Microbial Stress Tolerance for Biofuels. Microbiology Monographs.

[65] Ciobanu C., Tucaliuc A., Cascaval D., Turnea M., Galaction A.I. (2019). Correlation between aeration and ergosterol production by yeasts. Environ. Eng. Manag. J..

[66] Pupyshev A.B., Klyushnik T.P., Akopyan A.A., Singh S.K., Tikhonova M.A. (2022). Disaccharide trehalose in experimental therapies for neurodegenerative disorders: Molecular targets and translational potential. Pharmacol. Res..

[67] Singh L., Sharma S.C., Rai J. (2023). Role of Hal5p protein kinase under ethanol stress in *Saccharomyces cerevisiae*. Appl. Biol. Chem. J. (TABCJ).

[68] Lei J., Zhao X., Ge X., Bai F. (2007). Ethanol tolerance and the variation of plasma membrane composition of yeast floc populations with different size distribution. J. Biotechnol..

[69] Martinez-Munoz G.A., Kane P. (2008). Vacuolar and Plasma Membrane Proton Pumps Collaborate to Achieve Cytosolic pH Homeostasis in Yeast. J. Biol. Chem..

[70] Auesukaree C. (2017). Molecular mechanisms of the yeast adaptive response and tolerance to stresses encountered during ethanol fermentation. J. Biosci. Bioeng..

[71] Westergard L., True H.L. (2014). Wild yeast harbour a variety of distinct amyloid structures with strong prion-inducing capabilities. Mol. Microbiol..

[72] Yona A.H., Frumkin I., Pilpel Y. (2015). A relay race on the evolutionary adaptation spectrum. Cell.

[73] Fabrizio P., Garvis S., Palladino F. (2019). Histone Methylation and Memory of Environmental Stress. Cells.

[74] Zhao X.Q., Bai F.W. (2012). Zinc and yeast stress tolerance: Micronutrient plays a big role. J. Biotechnol..

[75] Ramos J., Ariño J., Sychrová H. (2011). Alkali-metal-cation influx and efflux systems in nonconventional yeast species. FEMS Microbiol. Lett..

[76] De Nadal E., Ammerer G., Posas F. (2011). Controlling gene expression in response to stress. Nat. Rev. Genet..

[77] Tanino T., Sato S., Oshige M., Ohshima T. (2012). Analysis of the stress response of yeast *Saccharomyces cerevisiae* toward pulsed electric field. J. Electrost..

[78] Causton H.C., Ren B., Koh S.S., Harbison C.T., Kanin E., Jennings E.G., Lee T.I., True H.L., Lander E.S., Young R.A. (2001). Remodeling of yeast genome expression in response to environmental changes. Mol. Biol. Cell.

[79] Saccharomyces Genome Database. https://www.yeastgenome.org/.

[80] Horak C.E., Snyder M. (2002). Global analysis of protein expression in yeast. Funct. Integr. Genomicc..

[81] Zahrl R.J., Prielhofer R., Burgard J., Mattanovich D., Gasser B. (2023). Synthetic activation of yeast stress response improves secretion of recombinant proteins. N. Biotechnol..

[82] Engelberg D., Perlman R., Levitzki A. (2014). Transmembrane signaling in *Saccharomyces cerevisiae* as a model for signaling in metazoans: State of the art after 25 years. Cell Signal..

[83] Rødkaer S.V., Faergeman N.J. (2014). Glucose- and nitrogen sensing and regulatory mechanism in *Saccharomyces cerevisiae*. FEMS Yeast Res..

[84] Kang X.X., Wang Q.Q., Chi Z., Liu G.L., Hu Z., Chi Z.M. (2021). The GATA type transcriptional factors regulate pullulan biosynthesis in *Aureobasidium melanogenum* P16. Int. J. Biol. Macromol..

